# The (cost-)effectiveness of an individually tailored long-term worksite health promotion programme on physical activity and nutrition: design of a pragmatic cluster randomised controlled trial

**DOI:** 10.1186/1471-2458-7-259

**Published:** 2007-09-21

**Authors:** Suzan JW Robroek, Folef J Bredt, Alex Burdorf

**Affiliations:** 1 Department of Public Health, Erasmus MC, University Medical Center Rotterdam, PO Box 2040, 3000 CA Rotterdam, The Netherlands; 2 LifeGuard Inc., PO Box 1366, 3500 BJ Utrecht, The Netherlands

## Abstract

**Background:**

Cardiovascular disease is the leading cause of disability and mortality in most Western countries. The prevalence of several risk factors, most notably low physical activity and poor nutrition, is very high. Therefore, lifestyle behaviour changes are of great importance. The worksite offers an efficient structure to reach large groups and to make use of a natural social network. This study investigates a worksite health promotion programme with individually tailored advice in physical activity and nutrition and individual counselling to increase compliance with lifestyle recommendations and sustainability of a healthy lifestyle.

**Methods/Design:**

The study is a pragmatic cluster randomised controlled trial with the worksite as the unit of randomisation. All workers will receive a standard worksite health promotion program. Additionally, the intervention group will receive access to an individual Health Portal consisting of four critical features: a computer-tailored advice, a monitoring function, a personal coach, and opportunities to contact professionals at request. Participants are employees working for companies in the Netherlands, being literate enough to read and understand simple Internet-based messages in the Dutch language.

A questionnaire to assess primary outcomes (compliance with national recommendations on physical activity and on fruit and vegetable intake) will take place at baseline and after 12 and 24 months. This questionnaire also assesses secondary outcomes including fat intake, self-efficacy and self-perceived barriers on physical activity and fruit and vegetable intake. Other secondary outcomes, including a cardiovascular risk profile and physical fitness, will be measured at baseline and after 24 months.

Apart from the effect evaluation, a process evaluation will be carried out to gain insight into participation and adherence to the worksite health promotion programme. A cost-effectiveness analysis and sensitivity analysis will be carried out as well.

**Discussion:**

The unique combination of features makes the individually tailored worksite health promotion programme a promising tool for health promotion. It is hypothesized that the Health Portal's features will counteract loss to follow-up, and will increase compliance with the lifestyle recommendations and sustainability of a healthy lifestyle.

**Trial registration:**

Current Controlled Trials ISRCTN52854353.

## Background

### Cardiovascular disease

Cardiovascular disease (CVD) is the leading cause of disability and mortality in most Western countries. CVD causes nearly half of all deaths in Europe (49%) [1]. Major modifiable risk factors for CVD include smoking, alcohol use, low physical activity (PA), and poor nutrition. The prevalence of several risk factors is very high, most notably low PA and poor nutrition (low fruit and vegetable consumption and high saturated fat intake).

According to a survey in European Union countries in 2002, 56% of the Dutch population over 15 years was insufficiently physically active for health [2]. The Dutch recommendation on PA stipulates that an adult should engage in PA of at least moderate intensity for at least 30 minutes a day on five days a week, and preferably every day in order to obtain health benefits [3]. In 2006, about half of the Dutch adults (25–55 year) met this recommendation [4]. In order to improve physical fitness it is recommended to engage in PA of vigorous intensity for at least 20 minutes on at least three days a week [3]. Exercise capacity has been found to be a powerful predictor of mortality [5]. It has been estimated that the life expectancy for people with low PA levels at age over 50 is 1.4 years less than for people with moderate PA levels and even 3.8 years less than for people with high PA levels [6].

The results of a recent meta-analysis on cohort studies indicate that fruit and vegetable consumption is inversely associated with the occurrence of coronary heart disease. The risk of coronary heart disease decreased by 4% for each additional portion of fruit and vegetables per day [7]. In the last representative Dutch food intake survey in 1997/1998 less than a fourth of the Dutch population met the recommendation for vegetable (200 grams a day) and fruit intake (200 grams a day) [8]. Regarding saturated fat intake, only 9% of the Dutch adult population met the recommendation (a maximum of 10 percent of energy intake as saturated fat) in 1997/1998 [9]. A high intake of saturated fat increases the risk of coronary heart disease [10].

The imbalance between PA and nutrition is an important cause of overweight and obesity, which in turn are important risk factors for CVD [11]. In the Netherlands self-reported overweight (body mass index ≥ 25) in adult men increased from 37% in 1981 to 51% in 2004, and in adult women from 30% in 1981 to 42% in 2004 [12].

### Worksite health promotion

In the prevention of cardiovascular disease, lifestyle behaviour changes are of great importance. Worksites have specific features that make them a promising place for health promotion. Worksites offer an efficient structure to reach large groups, enable the introduction of social support, and make use of a natural social network for peer support [13,14].

Literature shows contradictory results of randomised controlled trials (RCTs) on worksite health promotion programmes (WHPPs). A recent systematic review concluded that there is strong evidence for effectiveness of WHPP, based on two RCTs with a small effect on exercise behaviour and on energy expenditure [15]. However, another review on worksite PA programmes reported a small average effect size of 0.04 (95% CI -0.04–0.12) based on RCTs on self-reported level of PA 1–144 months after the intervention ceased [13]. A third review on environmental and policy interventions presented preliminary evidence that combined health education, screening, counselling, peer support, and access to (on-site) exercise equipment had positive effects on fitness levels, frequency of exercise, cholesterol levels, and systolic blood pressure. Several randomised studies on point-of-purchase nutrition interventions, some in worksites, showed positive effects on fruit and vegetable consumption, self-reported fat intake, cholesterol, and body weight but other studies have failed to corroborate these findings [16].

The overall picture emerges that WHPP may increase PA and improve nutritional intake among targeted groups, depending on the critical features of the interventions. Amongst others, as success factors of WHPP have been identified: (1) interventions tailored to the individuals' readiness for exercise adoption, (2) programmes that integrate specific components (nutrition, smoking, PA) into a combined approach, and (3) linking individual approaches to environment and policy conditions [17]. Marcus and colleagues showed that workers receiving self-help exercise promotion material tailored to the individual's readiness were significantly more likely to have increased exercise [18]. An individualized approach of high risk employees within the framework of a comprehensive program proved to be a critical feature of worksite interventions [19]. Recent studies have shown that web-based education tailored to personal characteristics may increase fruit and vegetable consumption and PA level, and decrease fat intake. In these interventions people received personalized feedback and advice that directly matched their individual behaviour, motivation, perceived (dis)advantages, and self-efficacy beliefs [20]. Based on results of their study on email messages to promote health behaviours, Franklin et al. suggest that emails may contribute tot the effective deliverance of health promotion programmes [21].

In contrast, three factors have been identified as greatest risks for ineffective WHPP: (1) a low, selective participation, (2) lack of adherence to the WHPP, and (3) an intervention effort too short for sustainable change in behaviour [13,15,16]. In several worksite studies intervention and evaluation periods were too short to determine the sustainable impact of environmental conditions [16].

In conclusion, previous WHPPs have shown contradictory results. Studies are needed on a WHPP that counteracts the three main factors for ineffectiveness.

In the study protocol described in this article, a long-term WHPP will be evaluated that adds the following four critical features to a traditional WHPP: (1) a computer-tailored advice on PA and diet (to increase awareness and adherence to the WHPP) (2) insight in progress over time on health-related behaviours (to increase adherence to the WHPP, compliance with the lifestyle recommendations and sustainability of a healthy lifestyle), (3) continuous feedback and support through monthly e-mails (personal coach) for 12 months (to increase adherence to the WHPP and compliance with and sustainability to the lifestyle recommendations), and (4) opportunities to seek personal advice from a variety of professionals (to increase adherence to the WHPP).

### Objectives

The aim of this pragmatic cluster randomised controlled trial is to evaluate the cost-effectiveness of a new investigator-driven WHPP with individually tailored advice in PA and nutrition and individual counselling to increase compliance with the lifestyle recommendations and sustainability of a healthy lifestyle.

## Methods/Design

### Study design and population

**Figure 1:**
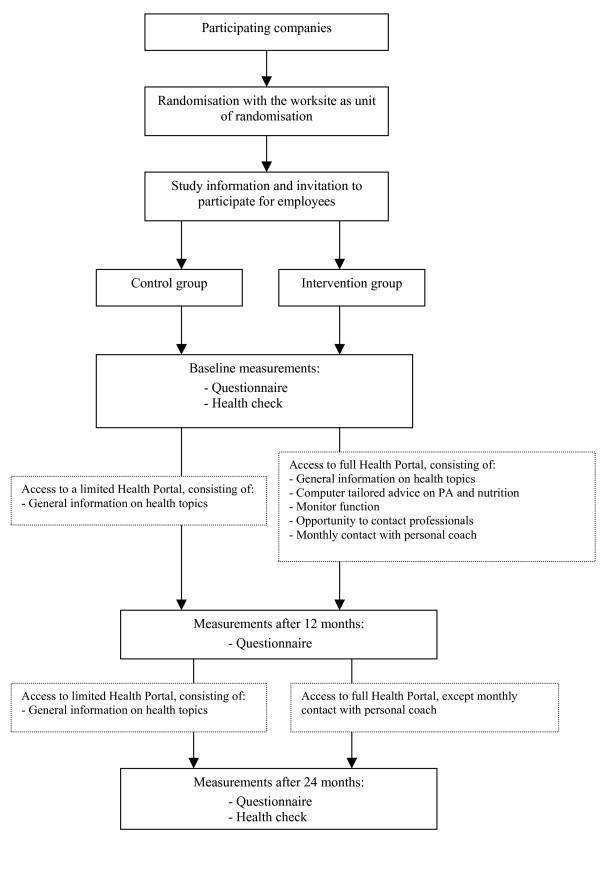
Flow of participants through the trial.

The study is a single blind pragmatic cluster randomised controlled trial with the worksite as the unit of randomisation. The (evaluation of the) intervention is targeted at the individual level. The study population consists of workers in companies that offer a standard WHPP to their employees, as provided by an organisation specialized in health management (LifeGuard Inc., Utrecht). Eligibility criteria for individual workers in the study are: 1) paid employment, 2) working at least 12 hours a week, and 3) being literate enough to read and understand simple e-mail and Internet-based messages in the Dutch language. All participants are blinded to the type of intervention. Data collection starts in September 2007 and will continue until August 2010. Participants will be requested to fill out a questionnaire at baseline and after 12 and 24 months. A physical examination (the 'health check') will take place at baseline and after 24 months. The study design and participant flow are shown in Figure 1. The Medical Ethics Committee of Erasmus MC, University Medical Center Rotterdam, the Netherlands, has approved the study protocol.

### Randomisation

Within each company, units will be randomised by a researcher not involved in the study, based on a table of random numbers (SAS command Ranuni). Within each company, worksites with comparable work activities and a comparable number of workers will be randomly allocated to the intervention or the control group. Subsequently, workers within each unit will be asked to participate in the study, presented as an evaluation study of different types of WHPP. All participants from one worksite will be randomised together rather than individually because individual randomisation may lead to contamination of the control group. Written informed consent at individual level is collected after agreement of the employer and randomisation at cluster level. Since it is deemed not possible within companies to withheld participation in a WHPP, workers within the control group will receive a standard WHPP.

### Standard program

The standard WHPP consists of:

1) A questionnaire to assess, among other things, PA level and fruit and vegetable intake.

2) A health check to assess weight, length, total blood cholesterol level, blood pressure, resting heart rate, body mass index (BMI), percentage of body fat, and predicted maximal oxygen uptake.

3) Advice of the provider's personnel, based on the outcomes of the questionnaire and health check. In addition, workers with a high total cholesterol level or high blood pressure will be referred to their general practitioner.

4) Access to a restricted part of the Health Portal on Internet, containing general information on health and health-related behaviours. The individual results on the questionnaire and health check are also retrievable through this web site.

### Intervention

On top of the standard WHPP the intervention group will have full access to the personalized Health Portal on Internet. The Portal contains four critical features: a computer-tailored advice, a monitoring function, a personal coach, and opportunities to contact professionals at request.

#### Computer-tailored advice

Participants will receive a computer-tailored advice to increase awareness of their lifestyle [22]. Awareness is found to be an important mediator of participation in health promotion programmes [23]. The benefits of computer tailoring is attributed to the fact that individualized feedback commands greater attention, is processed more intensively, contains less redundant information, and is appreciated better than the provision of general documentation [24].

After baseline measurements the participant in the intervention group will receive an email with the notification that a personal advice is available on the Health Portal. Considering the answers on the questionnaire filled out at baseline a personal advice on PA, fruit and vegetable consumption, and fat intake will be generated. The advice aims to increase adherence to the intervention and to motivate the participant to engage in PA and a healthy diet, and consists of the following parts:

1) Personal feedback: feedback on to what extent the recommendations on PA and dietary intake are met.

2) Action feedback: feedback on the specific barrier attributed by the participant as most important to not meeting the recommendations. The advice also contains opportunities to link to further information on the Health Portal and provides tips on how to meet the guidelines.

**Figure 2:**
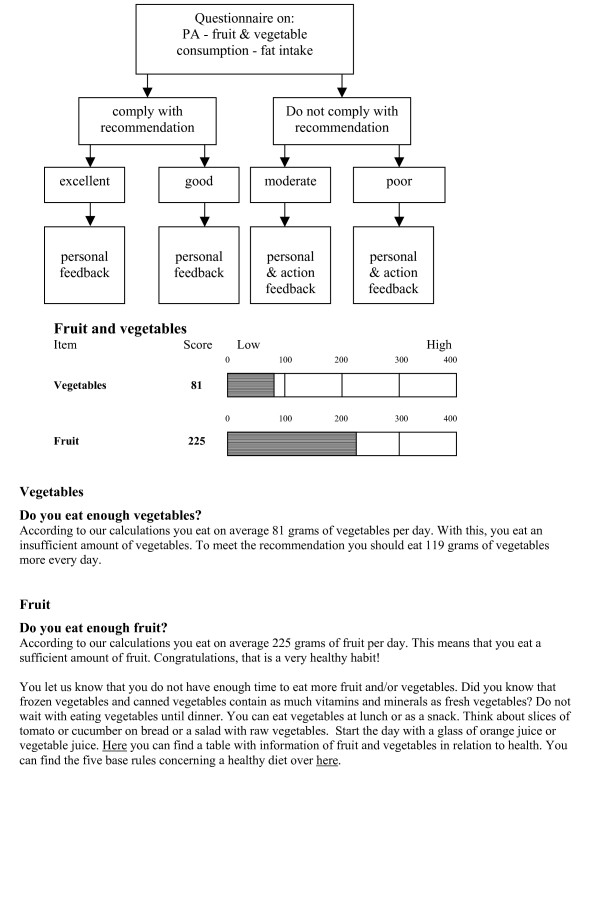
Example of the flow to a computer tailored advice.

If one meets the recommendations, only the personal feedback is provided. See Figure 2 for an example of the computer tailored advice.

#### Monitoring function

Second, a monitoring function is integrated in the Health Portal to increase adherence to the intervention programme and consequently increase compliance with the lifestyle recommendations and sustainability of healthy behaviour. Recent studies have shown that people who want to change their lifestyle should be encouraged to regularly monitor their progress in adopting a new behaviour [25,26].

**Figure 3:**
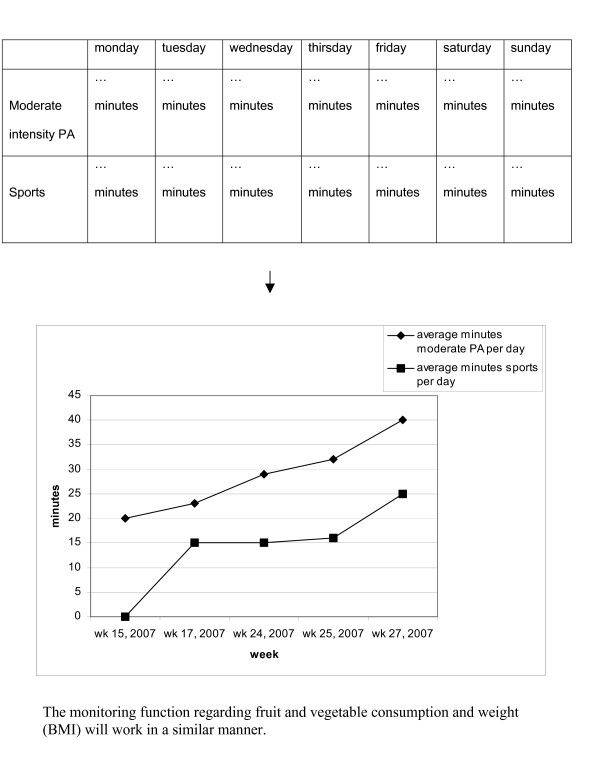
Example of the flow to a progress chart (monitoring function).

With the monitor function individual progress charts on self-reported weight, BMI, PA, and fruit and vegetable intake will be generated. The results of the baseline measurements will be used as starting point. The frequency of the use of this monitoring function is at the discretion of the participant. After 12 months the progress will be evaluated and communicated as part of the intervention. See Figure 3 for an example of a progress chart.

#### Personal Coach

The third critical feature of the Portal is a personal coach who will give continuous feedback and support through monthly emails. As like the monitoring function, this feature is part of the Health Portal to increase adherence to the intervention programme and to motivate participants to comply with the lifestyle recommendations and/or to maintain a healthy lifestyle. A previous study found promising results of individualized, interactive support for behaviour change on lifestyle [27].

Three groups of participants will be distinguished, based on Health Portal use and lifestyle. First, participants who do meet the recommendations on PA and/or fruit and vegetable intake at baseline will receive automatically generated emails with support to maintain their healthy lifestyle. Second, those not meeting the recommendation and not using the Health Portal will receive a reminder, a single question whether they have changed their behaviour and an invitation to use the monitoring function. Last, participants who use the monitoring function, but did not meet the recommendation at baseline, will receive a personal, not automatically generated, email with feedback on the data imported in the monitor.

If the participant does not want to receive the monthly emails, he can indicate this on the Health Portal.

#### Contact with professionals

The fourth feature is the opportunity to seek personal advice from a variety of professionals. By sending a message via the Health Portal participants can consult several experts such as a personal coach, a physiotherapist, or a dietician. This function is added to the Portal to increase adherence to the intervention programme.

The complete Health Portal will be offered for 12 months. After 12 months the monthly contact by a personal coach will be terminated, but access to the Portal will remain throughout the project.

### Measurements

#### Primary outcomes

##### Physical activity

PA level will be assessed by the self-administered short version of the International Physical Activity Questionnaire (IPAQ) [28]. The IPAQ consists of seven open-ended questions providing information on the time spent on walking, moderate- and vigorous intensity activities and in sitting in the past seven days. Participants will be instructed to refer to all domains of PA. Frequency per week and duration per day spent on the specific activity will be assessed. Concerning sitting, only duration per day will be assessed.

Both categorical and continuous indicators of PA will be calculated. By multiplying the metabolic-equivalent (MET) intensity for each activity with the weekly duration (in minutes) spent on each activity, the continuous measure (MET-minutes per week) will be calculated as recommended in the IPAQ scoring protocol [28]. The Dutch national guideline for PA stipulates that an adult should engage in PA of at least moderate intensity for at least 30 minutes a day on five days a week, and preferably every day [3]. As a categorical measure, compliance with the recommended amount of PA is defined by spending a total of at least 150 minutes on walking, moderate-intensity and vigorous-intensity PA per week [29]. In addition, compliance with the recommendation of vigorous PA will be assessed by examining if one does engage in vigorous PA on at least 3 days a week for at least 20 minutes on these days.

##### Fruit and vegetables

Fruit and vegetable intake will be assessed by means of a self-administered nine-item validated Dutch Food Frequency Questionnaire [30]. The questionnaire consists of seven items on fruit consumption and two items on vegetable consumption. First, participants will be asked to indicate on how many days during the last month they ate or drank the most often consumed fruit and vegetables in the Netherlands (i.e. apples, citrus fruit, cooked vegetables, etc.). Answer categories vary from 'never or less than once a month' to '7 days a week'. For all answers except 'never or less than once a month' a closed question follows in which one is asked to indicate the number of serving spoons, pieces, or units of juice consumed on such a day.

Total fruit consumption and total vegetable consumption will be calculated in grams. The total scores will also be used to determine compliance with the recommendations of an average of 200 grams of fruit and 200 grams of vegetables a day.

#### Secondary outcomes

##### Self-efficacy

Self-efficacy concerning PA and fruit and vegetable intake will be determined by asking how confident participants are to engage in PA and fruit and vegetable consumption in the next month, rated on a Likert scale between 1 (certainly) and 5 (certainly not) [31].

##### Perceived barriers

Perceived barriers concerning PA and fruit and vegetable intake will be assessed, by asking for the most important barrier to engage in these behaviours. The question on barriers to engage in PA has the following answer categories: not enough time/too busy, do not enjoy sports, too expensive, tired, fear of injury, no facilities at home, no facilities in direct environment, lack of a partner to exercise with, health problems, unsafe environment, and no barriers. The question on barriers concerning fruit and vegetable intake has the following categories: not enough time/too busy, not tasty, too expensive, no facilities at work to buy fruit and/or vegetables, no availability in the shops in the home environment, and no barriers.

##### Fat intake

Fat intake will be assessed by means of a self-administered 35-item validated Fat list covering 19 (groups of) food products. Participants will be asked about the frequency of food items during the last month with fixed categories. For each of the 19 categories a fat score, ranging from 0 (lowest fat intake) to a maximum varying from 3 to 5 points (highest fat intake), will be determined. Scores on the Fat list are presented in points instead of grams of fat, as only the most important saturated fat sources were included in the questionnaire [32]. A total fat score (range 0 – 80) will be calculated by adding up the 19 category fat scores.

##### Cardiovascular risk profile and physical fitness

The risk profile for cardiovascular events will be assessed by the SCORE (Systematic Coronary Risk Evaluation) system, taking into account the following risk factors: sex, age, smoking, total cholesterol level, and systolic blood pressure [33]. Sex, age, and smoking will be assessed by questionnaire. During a health check total blood cholesterol level will be determined in non-fasting blood through a finger prick (Accutrend GC, Roche Company, Mannheim, Germany). Systolic blood pressure will be measured with a fully automated sphygmomanometer (Omron M4-I, Omron HealthCare Europe BV, Hoofddorp, the Netherlands). With this sphygmomanometer resting heart rate will be measured as well.

In addition to these measures length and body weight will be measured to determine BMI (kg/m^2^). Waist circumference and thickness of three skin folds (i.e. men: pectoralis major, abdomen, quadriceps; women: triceps, crista iliaca, quadriceps) will be measured to calculate body fat percentage.

A submaximal exercise test on a bicycle ergometer will be conducted to predict maximal oxygen uptake, according to the American College of Sports Medicine's (ACSM) protocol, using three-minutes stages and terminates at approximately 80% of the age predicted maximum heart rate. The initial test workload will depend on age, sex, and exercise status. The maximum number of workload stages is four, and the minimum test time is nine minutes. Participants will be asked to pedal with a frequency of 60 revolutions per minute (rpm). During the test, heart rate will be recorded, and used to predict maximal oxygen uptake (Vo_2_max). All the physical measurements will be done according to the guidelines for exercise testing of the ACSM [34].

#### Confounding variables

Possible confounders include demographics, smoking behaviour, and general health. The demographic variables of importance are age, ethnicity, educational level, sex, and marital status. Smoking is defined as current smoking status. General health will be assessed using the Short Form-12 questionnaire [35]. In addition, some questions on occupation will be asked: job title, years in current job, days and hours of work (including overtime work), main job requirements (physical or mental), and working conditions [36]. During a worksite visit, environmental determinants will be assessed, especially available resources in the company to provide and sustain healthy behaviour (e.g. fitness room, financial compensation for membership of sport/fitness club, stairs, fruit and vegetables in canteen). Further, participants will be asked if they have Internet availability at home.

### Process evaluation

#### Participation

In the non-response analysis the following characteristics of (non)participants will be considered: age, sex, education, job title. In addition, enterprise size (number of employees) and history of health promotion activities in the company will be considered.

#### Adherence and Sustainability

Adherence to the intervention programme will be analysed in relation to compliance with lifestyle recommendations on PA and on fruit and vegetable intake, and in relation to sustainability of a healthy lifestyle. As markers of adherence to the intervention programme, the frequency of visiting the Health Portal, duration of stay on the Portal and frequency of contacts with professionals and personal coach will be registered. This will be done for the full Health Portal, as well as separately for the different parts of the Health Portal.

In addition, characteristics of participants (demographic variables, lifestyle at baseline, job, social support from colleagues and friends, and Internet availability at home) will be analysed as to subgroups with the best adherence to the intervention programme, compliance with the lifestyle recommendations, and sustainability of a healthy lifestyle.

### Cost-effectiveness evaluation

The cost-effectiveness analysis will be performed from a societal perspective as well as a company perspective. The following direct costs will be determined: cost price of the standard WHPP, costs for the Health Portal and direct costs of medical consumption. Direct costs of medical consumption will be based on frequency of contacts with a variety of health professionals and average remuneration fee, assessed by an adapted version of the Dutch Trimbos and iMTA Questionnaire on Costs Associated with Psychiatric Illness (TiC-P) [37]. The direct costs will be measured over the complete follow-up period of 24 months with annual questionnaires with 12-month recall.

The indirect costs will be based on assessment of days with loss of productivity at work due to health problems and productivity loss due to sickness absence, using the Dutch productivity and disease questionnaire (PRODISQ) [38]. The estimated days of productivity loss will be multiplied by the average wage per day for each worksite.

In a second step cost-effectiveness ratios will be calculated on two measures of health: general health and the SCORE risk profile for cardiovascular events.

### Sensitivity analysis

The sensitivity analysis will start with the analysis of the effectiveness of the intervention in specific subgroups, most notably those workers with a low physical activity level, with a low intake of vegetables and fruit, and with a high body mass index. A sensitivity analysis will also be performed on the individual cost-effectiveness ratios by means of bootstrapping. This part of the sensitivity analysis will be used to determine the minimum level of effectiveness required to make the Health Portal more cost-effective than the standard WHPP.

### Sample size

The assumptions for the power calculations were: an intra-cluster correlation of 0.05 (as observed in a previous cluster RCT in companies [39]), an average of 20 workers per cluster, a power of 80%, and a level of significance of 5% (one-sided). Under these assumptions, we anticipate to be able to detect a difference of at least 12% in prevalence between intervention and control group (e.g. primary outcome measure 30% compliance with the recommendation for PA up to 42%) with 350 workers with completed questionnaires assigned to the intervention. Without a noticeable intra-cluster variance the detectable difference will increase by 9%. With an initial participation of 70% and loss-to-follow-up of 30%, the cluster RCT should invite 2*700 workers.

### Statistical analysis

An intention-to-treat analysis will be used with last available information carried forward to missing data in subsequent measurements. A multilevel linear regression model with repeated measurements will be used (SAS proc Mixed) for continuous outcomes and a hierarchical logistic regression (SAS proc Genmod) for dichotomous outcomes. The effect of self-efficacy, environmental determinants, Portal use, and drop-out during follow-up on primary and secondary outcome measures will be evaluated for their potentially differential effects.

Although the discriminatory power will be limited, an analysis will be carried out as to which subgroups participate in the WHPP and the Health Portal and which subgroups have the best adherence to the lifestyle recommendations (age, sex, education, ethnicity).

In addition, preliminary analyses will be performed for workers with cardiovascular complaints and workers with obesity in order to evaluate whether these subgroups are more or less amendable to changing their lifestyle.

## Discussion

In this study protocol the design of a pragmatic cluster randomised controlled trial on worksite health promotion is presented. The study is designed to evaluate the (cost)effectiveness of an individually tailored long-term worksite health promotion programme on PA and nutrition. It is hypothesized that the unique combination of critical features (a computer-tailored advice, a monitor function, a personal coach, and the opportunity to contact professionals at request) counteracts the main factors for ineffective WHPP (lack of participation, adherence to the WHPP and sustainability), and leads to a change in lifestyle. By conducting an extensive process evaluation we gain insight into the effective elements of worksite health promotion. By registering several process variables it is possible to find out if participants with a higher adherence to the (separate parts of the) WHPP are more likely to comply with the lifestyle recommendations.

With the health check as starting point for the WHPP, it is aimed to increase participation. The Health Portal's critical features are aimed to counteract loss to follow-up, and increase adherence to the intervention programme, compliance with lifestyle recommendations, and sustainability of a healthy lifestyle. Because of the long-term follow-up, sustainability of healthy behaviour will be facilitated.

The cost-effectiveness of the extensive Health Portal will be compared to the cost-effectiveness of the standard WHPP.

In conclusion, this study evaluates a promising intervention on healthy behaviour and results will provide insight into cost-effectiveness and the effective elements of WHPP.

## Competing interests

SR declares that she has no competing interests. AB has advised LifeGuard Inc. with respect to validity and reliability of questionnaires on working conditions, work ability, medical consumption and productivity losses in past. FB is Research and Development director at LifeGuard Inc.

## Authors' contributions

AB and FB originated the idea for the study and its design. AB was responsible for acquiring the grant for the study. SR further developed the described intervention protocol and will be responsible for data collection, data analysis, and drafting of the final research report. The authors participated in discussing the design of the study and developing the research protocols. All authors read and approved the final manuscript.

## Pre-publication history

The pre-publication history for this paper can be accessed here:


